# The effect of an airflow restriction mask (ARM) on metabolic, ventilatory, and electromyographic responses to continuous cycling exercise

**DOI:** 10.1371/journal.pone.0237010

**Published:** 2020-08-11

**Authors:** João Francisco Barbieri, Arthur Fernandes Gáspari, Cassia Lopes Teodoro, Leonardo Motta, Luz Albany Arcila Castaño, Romulo Bertuzzi, Celene Fernandes Bernades, Mara Patrícia Traina Chacon-Mikahil, Antonio Carlos de Moraes

**Affiliations:** 1 Department of Sport Science, School of Physical Education, University of Campinas, Campinas, Brazil; 2 Endurance Performance Research (GEDAE-USP), School of Physical Education and Sport, University of São Paulo, São Paulo, Brazil; University of Bourgogne France Comté, FRANCE

## Abstract

This study analyzed the physiological adjustments caused by the use of the Elevation training mask® (2.0), an airflow restriction mask (ARM) during continuous exercise. Eighteen physically active participants (12 men and 6 women) were randomized to two protocols: continuous exercise with mask (CE-ARM) and continuous exercise without mask (CE). Exercise consisted of cycling for 20 minutes at 60% of maximum power. Metabolic variables, lactate, and gas concentration were obtained from arterialized blood samples at pre and post exercise. Continuous expired gases and myoelectric activity of the quadriceps were performed at rest and during the test. We observed no reduction in oxygen saturation in CE-ARM, leading to lower pH, higher carbon dioxide, and greater hematocrit (all p <0.05). The expired gas analysis shows that the CE-ARM condition presented higher oxygen uptake and expired carbon dioxide concentrations (p <0.05). The CE-ARM condition also presented lower ventilatory volume, ventilatory frequency, and expired oxygen pressure (p <0.05). No changes in electromyography activity and lactate concentrations were identified. We conclude that using ARM does not induce hypoxia and represents an additional challenge for the control of acid-base balance, and we suggest the use of ARM as being suitable for respiratory muscle training.

## Introduction

The benefits of hypoxic training are well documented in the literature and this type of training is known to act upon various body systems, including the central nervous, cardiorespiratory, and muscular systems [1,2]. Because training in high altitudes or using the normobaric hypoxia strategy is not easily accessible, other methods to simulate hypoxic training were developed in the last few years. The Elevation training mask^®^ is one of these products. Nowadays known as Training Mask^®^, this mask is characterized as an airflow restriction mask (ARM). In the marketing material for the product (trainingmask.com), suggestions are made comparing the ARM use and hypoxia exposure (see Training mask Works–Scientifically Proven Performance section). The site states that the use of the mask would increase the circulation Growth Hormone induced by hypoxic conditions [3]. The resistance to airflow would provide this hypoxia condition during breathing via an adjustable valve system that have different “altitude” levels (Elevation Training Mask 2.0). In addition, the ARM is postulated to increase respiratory muscle work leading to improvements in endurance performance, via respiratory muscle training (RMT), as shown in previous literature [4].

The effectiveness of ARMs is not well understood because of the limited number of studies, and limited access to more direct and precise physiologic measurements. Studies using ARMs in long-term training (6 weeks or more) found no major changes in oxygen saturation and cardiovascular adaptations such as maximum oxygen intake $\left(\dot{V}O_{2\;\max}\right)$ or time to exhaustion test [5,6]. Porcari et al. [7] observed that aerobic performance improved after 6 weeks of training, which was attributed to the potential action of ARMs influencing the respiratory muscles, although there were no significant differences in the results of pulmonary function tests. Furthermore, Porcari et al [7] demonstrated that ARMs significantly improved $\dot{V}O_{2}$ and power output at the respiratory compensation point and ventilatory threshold. However, the metabolic and neuromuscular responses to exercise in the presence or absence of ARMs were not investigated by these authors, and remain unknown.

In a randomized, counterbalanced, and within-subjects study, Romero-Arenas [8] demonstrated that the use of ARMs during a maximal incremental exercise test on a cycle ergometer reduced maximal power and lactate concentration. The use of ARMs in the test did not significantly affect oxygen saturation (SaO_2_) in the muscle, but did increase the delivery of O_2_ to the frontal cortex measured with near-infrared spectroscopy. The authors attributed this phenomenon to a hypercapnic state induced by the mask, but did not measure this variable (pCO_2_) in the blood. Granados et al. [9] also investigated the use of ARM, showing that acute treadmill exercise using a modified version of ARM reduces ventilation (VE) and respiratory rate (RR), which can lead to an increase in CO_2_ blood level, although this variable was not directly assessed in the study.

The purpose of this study was to compare blood gases, blood acid-base balance, ventilatory, and electromyographic (EMG) responses during continuous acute cycling exercise using ARM. We hypothesized that ARMs are effective in inducing a decrease in SaO_2_ when measured directly in the blood, as well as an increase in blood CO_2_. We hypothesized that the mask use will cause changes in metabolites and EMG signals due to alterations in acid-basic balance variables.

## Materials and methods

### Participants

The study included 18 healthy subjects (12 men and 6 women; age, 25.1 ± 4.6 years; BMI, 22.4 ± 2 kg/cm^2^; height, 170 ± 10 cm; $\dot{V}O_{2\max}$, 46.8 ± 4.0; maximum power, 247 ± 44 watts) who were physically active (engaged regularly in at least 3 days of physical activity per week). Before the study, the participants were informed about the protocols, possible risks and benefits, and the schedule of activities. The participants were instructed not to perform strenuous physical exercises for 48 hours and not to consume alcohol or stimulant drugs for 24 hours before the tests. Also all participants was instructed to maintenance the supplements that their used and avoid new supplements. The study was approved by the Research Ethics Committee of the University of Campinas (Protocol No. 1.376.230) and was conducted in accordance with the Declaration of Helsinki.

### Experimental design

Subjects completed six visits to the laboratory. The first visit was intended to familiarize subjects with the environment, procedures, and exercise protocols. All participants signed the consent form during the first visit. In the second and third visits, a maximal incremental exercise test was performed on a cycle ergometer in order to obtain the maximum power output (Pmax). Two maximal incremental exercise tests were carried out to improve the reliability of the evaluation. The highest Pmax in the two tests was used for training intensity prescription.

The Pmax was used for exercise prescription in the experimental sessions. The fourth visit was intended to familiarize the subjects with the exercise protocol using ARMs. During visits five and six, the participants performed a continuous exercise bout with ARMs (CE-ARM) or a continuous exercise without ARMs (CE) in a crossover, counterbalanced, randomized and within-subject design.

The maximal incremental exercise tests and the two exercise protocols were performed on the same cycle ergometer (RacerMate®, CompuTrainer^TM^, Seattle, USA) at the same time of day using the same seat, handlebar, and gait settings. The ARMs were configured to simulate an altitude of 15,000 feet (4,572 m) in the CE-ARM condition. The CE condition wore the mask to maintain the same dead space volume but did not use the valves to allow normal VE. The ARM configuration, test protocols, and interval between visits (at least 48h) were determined previously using a pilot study in our laboratory.

### Experimental session

For the EMG analysis, electrodes were placed in the quadriceps muscles. After that, the participants remained seated for 10 min, and blood was collected from the earlobe and the distal phalanges of the fingers for metabolic (see Table 1) and blood gas analyses. Participants were positioned on the cycle ergometer at the beginning of the test, and expired gases were collected for seven min at rest as baseline parameters. After this collection, the test began.

**Table 1:** Metabolic variables.

Variables	Conditions	Pre	Post	Δ (%)
**pH**	CE	7,42 ± 0.01	7,33 ± 0.03*	1.21
	CE-ARM	7,42 ± 0.001	7,29 ± 0.06* †	1.71
**cHCO**_**3**_ **(mmol/L)**&	CE	22,31 ± 1,88	17,96 ± 2,14	19.43
	CE-ARM	21,76 ± 1,66	17,62 ± 3,25	19.02
**HHb (%)**&	CE	1,87 ± 1,01	3,14 ± 1,25	67.92
	CE-ARM	1,77 ± 0.81	3,79 ± 1,55	114.1
**BE (mmol/L)**&	CE	-1,54 ± 1,52	-6,98 ± 2,16	353.2
	CE-ARM	-1,98 ± 1,27	-8,25 ± 3,71	319.61
**Lactate (mmol/L)**&	CE	1,66 ± 0.51	7,71 ± 3,31	364.4
	CE-ARM	1,53 ± 0.45	7,90± 2,92	416.34
**pO**_**2**_ **(mmHg)**&	CE	85,15 ± 7,47	80.62 ± 6,32	5.32
	CE-ARM	82,43 ± 4,04	79,96 ± 5,39	3.03
**pCO**_**2**_ **(mmHg)**	CE	35,35 ± 3,19	34,76 ± 3,33	1.66
	CE-ARM	34,61 ± 3,25	37,39 ± 4,06 * †	8.03
**Hct (%)**	CE	44,23 ± 4,28	46,93 ± 4,46*	6.10
	CE-ARM	45,01 ± 4,37	48,99 ± 4,78* †	8.84
**SaO**_**2**_ **(%)**&	CE	98,09 ± 1,03	96,81 ± 1,26	1.30
	CE-ARM	98,19 ± 0.84	96,16 ± 1,58	2.06

Abbreviations: BE, base excess; CE-ARM, continuous exercise with mask; CE, continuous exercise without mask; Hct, hematocrit; HCO3, bicarbonate concentration; HHb, deoxyhemoglobin; SaO2, oxygen saturation; pO2, partial oxygen pressure; pCO2, and partial carbon dioxide pressure.

Data are expressed as Mean ± SD.

* interaction effect between time for the same condition (p <0.05). & difference between time (p <0.05).

† difference between conditions (p <0.05).

The cycling load of the CE-ARM and CE conditions was set at 60% of the Pmax, and was held constant throughout the test at a cadence of 65–75 revolutions per minute (rpm). Both experimental sessions lasted 20 min.

Expired breath-to-breath gases and the EMG activity of quadriceps muscles were collected during all the test. Blood samples were drawn from the distal phalanges of the fingers immediately and at three, five, and seven min after exercise. Arterialized blood was drawn from the left ear lobe for metabolite analysis at the end of the test.

### ARM adaptation piece to gas analyzer

**Fig 1:**
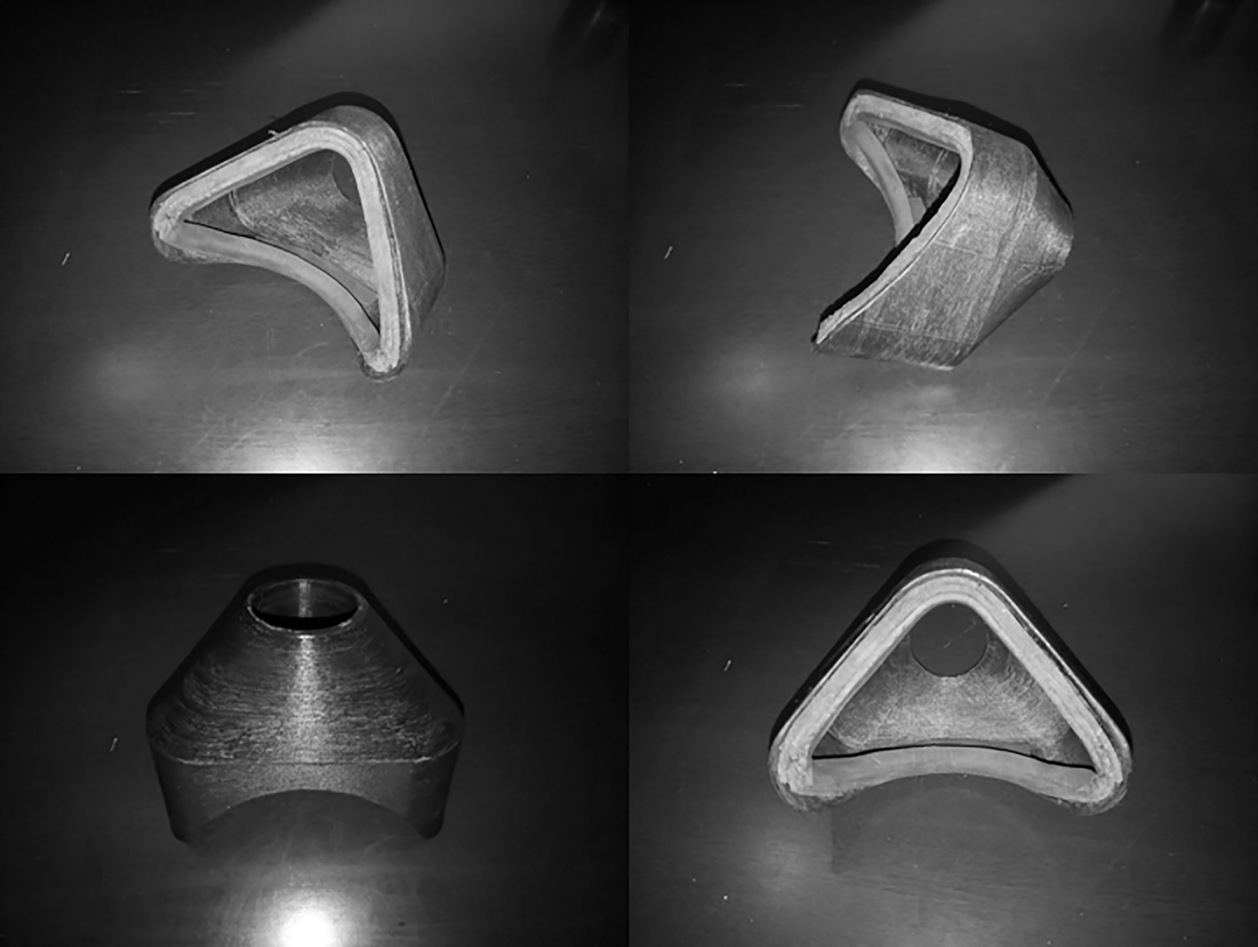
Piece to connect the ARM to the gas analyser. The 3D piece was used to connect the ARM to the gas analyser. The complex ARM + 3D piece had a dead space of 350 ml.

ARM was coupled to the gas analyzer using a mouthpiece made with the polymer acrylonitrile-butadiene-styrene in an FDM-type 3D printer (RepRap Prusa Mendel I3, Sethi Indústria e Comércio de Produtos Eletrônicos, São Paulo, Brazil). The total volume of the ARM and mouthpiece was approximately 350 ml (see Fig 1). The experimental tests were performed in both conditions using the mouthpiece and ARM (no valves were used in the CE condition) to keep the same dead space volume between the two conditions. Neoprene was used on the inner edge of the mouthpiece to ensure proper sealing, and the mouthpiece was attached to the ARM using adhesive tape.

### Maximum incremental test

The test was performed on a cycle ergometer (CompuTrainer^TM^, RacerMate®, Seattle, USA) with continuous collection of gas exchange ($\dot{V}O_{2}$, $\dot{V}{\mathrm{CO}}_{2}$ and VE) using an automatic gas analyzer (CPX Ultima^TM^, Medgraphics®, Minnesota, USA). The calibration of the cycle ergometer and automatic gas analyzer was identical in all tests. The gear ratios and adjustments were identical in all tests and experimental sessions for both conditions. The participants performed progressive exercise to exhaustion. The load was considered maximum when at least two of the following criteria were met: 1. Plateau in oxygen uptake, defined as an increase of less than 2.1 ml/kg/min in oxygen volume ($\dot{V}O_{2}$) even with increased load; 2. Respiratory exchange ratio ≥ 1.1; and 3. Heart rate (HR) ≥ 90% of the maximum predicted by age (220 –age). These criteria were based on the study by Howley, Bassett, and Welch [10]. The test comprised a preliminary five-min rest and five-min warm-up to 50 W, followed by increments of 25 W every minute. The subjects were instructed to maintain a cadence of 70–75 rpm. When it was impossible to maintain the desired cadence, two reestablishment attempts were given. The test was discontinued when the participant failed to maintain cadence on the third attempt, even under strong verbal encouragement. The third failed attempt was considered physical exhaustion. Heart rate was recorded during the test using a cardio-frequency meter (Polar, Kempele, Finland).

### Blood collection and analysis

For analysis of the lactate concentration [Lac], 25μL blood samples were collected from the distal phalanges of the fingers by puncture with disposable lancets after cleaning the fingers with 70% alcohol. Blood was collected immediately before, immediately after, and at three, five, and seven min after the end of the exercise. The blood samples were immediately transferred to microtubes containing 25μL of 1% sodium fluoride and centrifuged for 10 min at 5,000 rpm (Centrifuge Excelsa Baby I, Fanem, São Paulo, Brazil). The supernatant plasma was harvested using an automatic pipette and stored at –80°C for further analysis. Lactate concentration was measured in a spectrophotometer (ELx800, Biotek®, Vermont, USA) using commercial kits (Biotecnica®, São Paulo, Brazil).

Blood (approximately 125 μl) was collected from the earlobe in capillary tubes (Roche, Diagnostics GmbH, Mannheim, Germany) before and immediately after the exercise to analyze the following blood gas variables: partial oxygen pressure (pO_2_), partial carbon dioxide pressure (pCO_2_), pH, deoxyhemoglobin (HHb), base excess (BE), hematocrit (Hct), bicarbonate concentration (HCO_3_), and oxygen saturation (SaO2). Ten minutes before the first blood collection, the earlobe was pre-warmed for 5 min to activate blood flow and facilitate absorption of a vasodilator cream (Finalgon; Laboratorios FHER, SA, Barcelona, Spain) [11]. When necessary, the cream was applied again before the second collect. Blood samples were immediately analyzed using a point-of-care blood gas analyzer (Cobas b 123 POC System; Roche Diagnostics GmbH, Mannheim, Germany).

### Electromyographic signal acquisition and processing

A Biopac EMG system model MP150 was used (Biopac System, Inc.; Santa Barbara, CA, USA) for EMG signal acquisition. This model has 16 channels, active bipolar electrodes (model TSD-150), and a common-mode rejection ratio of >95 dB. AcqKnowledge software version 3.8.1 (Biopac System, Inc., CA, USA) was used for signal analysis. The sample rate acquisition was 2000 Hz, and the bandpass filter was 20–500 Hz.

The surface electrodes were attached to the rectus femoris (RF), vastus lateralis (VL), and vastus medialis (VM) muscles with adhesive tape according to the recommendations of Surface EMG for Non-Invasive Assessment of Muscles [12]. The centers of the electrodes were kept at a fixed distance of two centimeters. A reference electrode was placed on the tibial tuberosity of the right leg of each participant. Hair and dead cells were removed from the skin to decrease impedance. After signal collection, the root mean square (RMS) of EMG signals (100 bursts) was calculated using the software AcqKnowledge version 3.8.1 (Biopac System, Inc., CA, USA). After that, data were extracted, and a specific routine created in the MATLAB (MathWorks) software was used to calculate the mean values per minute. The RMS result per minute was normalized by the RMS of the first minute of each protocol. The normalized signals of the RF, VM, and VL muscles were summed and presented as a single variable (SUM QF).

### Statistical analysis

The normality of the data was confirmed using the Shapiro-Wilk test. Two-way analysis of variance (ANOVA) was used for repeated measurements. The Tukey *post-hoc* test was applied to analyze the interaction effect between *condition* and *time* (pre and post) in ANOVA. The significant main effects of *time* and *condition* are indicated in the text. When appropriate, Tukey *post-hoc* test was used to examine the main effect of *time*. The STATISTICA software version 6.0 was used for all statistical analyses (StatSoft, Inc., Tulsa, OK, USA). Data are presented as mean and standard deviation (SD), and the significance level adopted for all comparisons was p<0.05.

## Results

### Metabolic variables

The results of the metabolic variables are shown in Table 1. The pH decreased in the CE and CE-ARM condition after exercise (CE p<0.01; CE-ARM p<0.01), and the decrease was larger in the CE-ARM condition (p<0.01). HCO_3_, HHb, BE, and [Lac] showed a main effect of time (HCO_3_ p<0.01; HHb p<0.01; BE p<0.01 and [Lac] p<0.01).

The pO_2_ showed a main effect of time (p = 0.05); as the concentration of pO_2_ pre-exercise was smaller than post-exercise for both conditions. The pCO_2_ remained unaltered in the CE condition and increased in the CE-ARM condition (p<0.01), and the difference between the conditions was significant (p<0.01). Hct increased in both conditions post-exercise (both at p = 0.05) and was higher in the CE-ARM condition (p<0.01). SaO_2_ did not present an interaction effect between the conditions (p = 0.08) but showed a main effect of time (p<0.01) and was lower after the test in both conditions.

### Gas analysis and ventilatory variables

**Fig 2:**
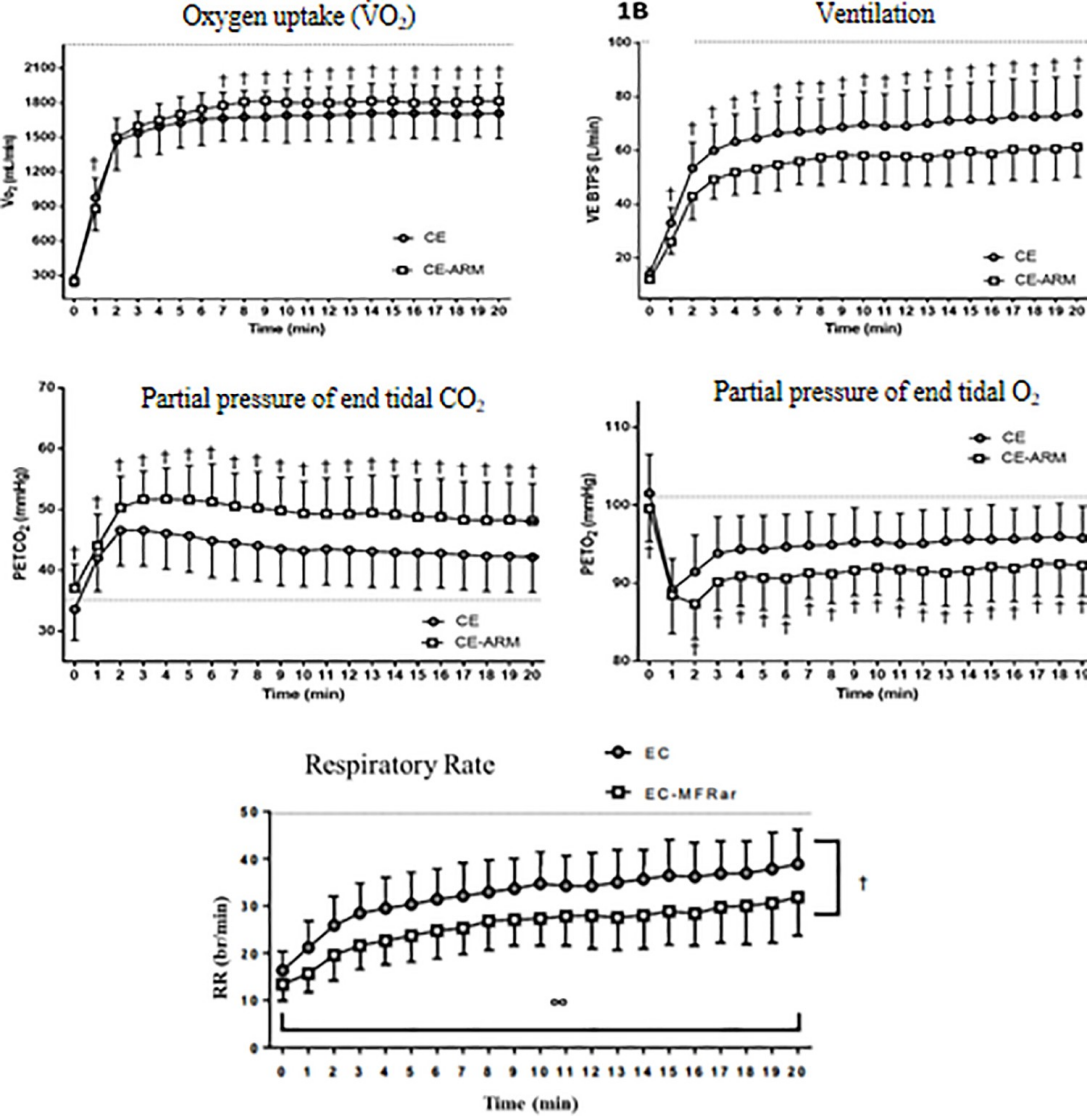
Abbreviations: CE-ARM, continuous exercise with mask; CE, continuous exercise without mask; PETCO_2,_ Partial pressure of end tidal CO_2_; PETO_2,_ Partial pressure of end tidal O_2;_ RR, Respiratory rate; $\dot{V}O_{2}$, Oxygen uptake and VE, Ventilation. Data expressed in Mean (Standard Deviation). † denotes a significant difference between conditions (CE x CE-ARM) identified by the Post Hoc (p <0.05). ∞ Denotes main effect of time identified by ANOVA (p <0.05).The dashed line indicates the maximum mean value reached in the incremental test.

A summary of ventilatory variables is presented in Fig 2. $\dot{V}O_{2}$ was higher in the CE condition in the first minute of the test (p<0.01) and higher in the CE-ARM condition from the seventh minute until the end of the test (all p<0.01) (Fig 2A). VE was lower in CE-ARM at all time points (p<0.01) (Fig 2B). The partial pressure of end-tidal CO_2_ (PETCO_2_) was higher in CE-ARM at all time points (p<0.01) (Fig 2C). The partial pressure of end-tidal O_2_ (PETO_2_) was lower in the CE-ARM condition before the test (p = 0.02) and from the second minute to end of the test (all p<0.01) (Fig 2D). The RR showed a main effect of condition (p<0.01) and time (p<0.01), and the *post-hoc* test indicated that this variable increased throughout the test. However, the *post-hoc* analysis found no interaction effect between the conditions (p = 0.24).

### Electromyographic variable

**Fig 3:**
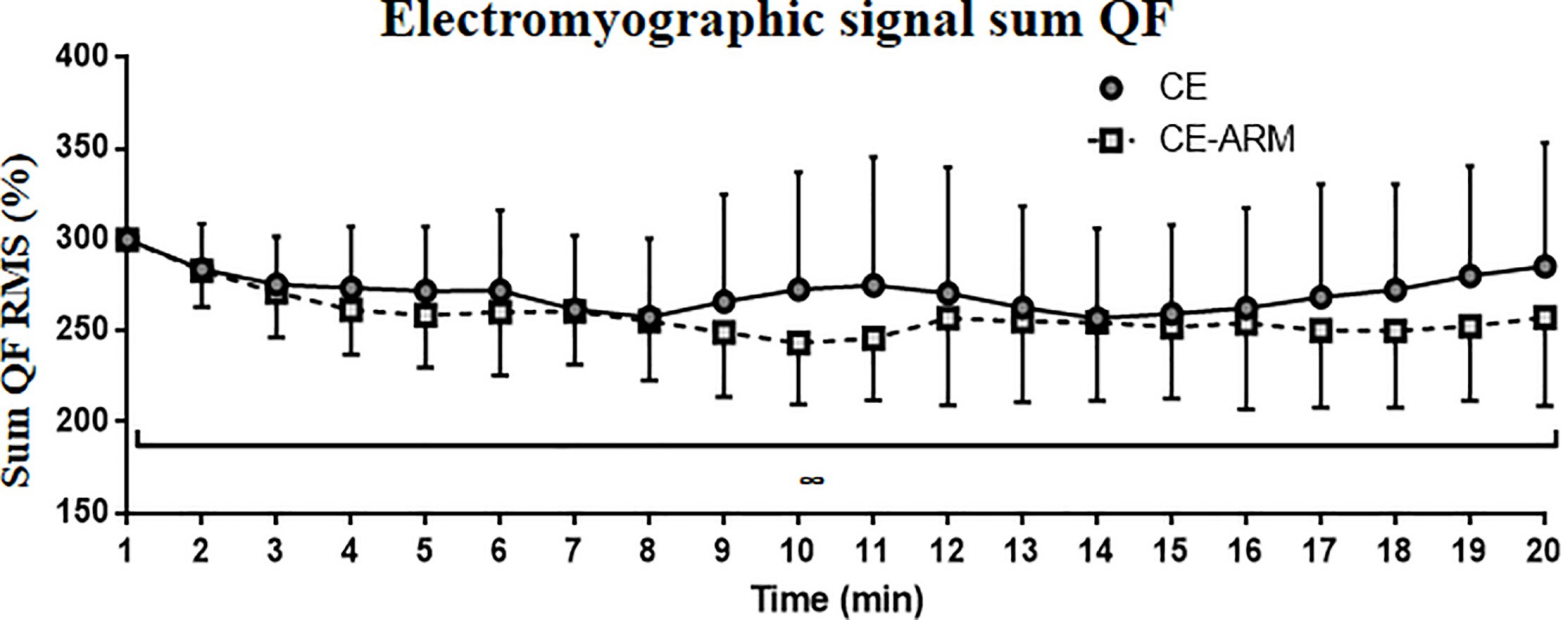
EMG response to the continues test. Abbreviations: CE-ARM, continuous exercise with mask; CE, continuous exercise without mask; Sum QF, the sum of the signals of the already normalized RF, VL and VM muscles. Data are expressed as Mean (Standard Deviation). ∞ Denotes main effect of time identified by ANOVA (p <0.05).

The analysis of SUM QF (Fig 3) data revealed that there was no interaction effect (p = 0.27) between condition and time (p<0.01). However, there was a main effect of time (p<0.05). The *post-hoc* analysis indicated that the main effect of time was not significant at any time point.

## Discussion

This study evaluated whether the use of ARMs in continuous aerobic exercises could simulate training in hypoxic conditions and cause metabolic and electromyographic changes. ARMs caused significant changes in ventilatory variables, with a decrease in VE and PETO_2_ and RR and an increase in O_2_ consumption and PETCO_2_. Moreover, there were significant changes in the metabolic profile with the use of ARMs relative to CE, evidenced by lower blood pH, higher blood CO_2_, and elevated hematocrit.

Our findings indicated that ARM did not induce hypoxia because the level of decrease in SaO_2_ was similar between the two conditions. Furthermore, the absence of hypoxia was evidenced by the lack of significant differences in pO_2_ values between the conditions. The other analyzed variables, as Lactate, corroborate that ARMs did not cause hypoxia. Hypoxic training causes pronounced changes in [Lac] when compared with normoxic training [1]. There were no significant differences in [Lac] between the conditions. In addition, it is known that myoelectrical activity is attenuated under hypoxic conditions [1], which did not occur in the present study, whereby there were no significant differences in SUM QF between the conditions.

ARM did not cause hypoxia, but significantly increased Hct. Hematopoietic changes due to exposure to hypoxic environments are considered essential for improving athletic performance [13]. Adaptations such as an increase in baseline Hct are observed in conditions involving chronic exposure to environments with low O_2_ saturation as a result of the action of the hormone erythropoietin [14]. However, the increase in Hct at the end of the exercise cannot be attributed to a hypoxic condition, and may be a mechanism to control acid-base balance because the pH was lower in the CE-ARM condition, even with an increase in HHb after exercise in both conditions. The increase in HHb suggests that hemoglobin serves to buffer excess hydrogen protons (H^+^) [15]. In addition, it is possible that the decrease in pH and the increase in pCO_2_ stimulated a sympathetic response for spleen contraction, releasing reticulocytes into the bloodstream, which could contribute to the increase in Hct [16].

The lower blood pH found in the present study was attributed to respiratory acidosis. The occurrence of metabolic acidosis was discarded because [Lac] was similar in both conditions after exercise, demonstrating that the additional source of H^+^ protons did not come from the active leg muscles. This is in agreement with non-differences in quadriceps muscles activation, since there was no significant differences in the electromyographic signal of SUM QF (p = 0.27).

Respiratory acidosis is common in some conditions such as chronic obstructive pulmonary disease [17], but this condition can be induced by some techniques such as prolonged expiration, as demonstrated in triathletes [11]. The impaired respiratory mechanics affect the acid-base balance and cause the accumulation of blood CO_2_. In this study, ARMs impaired respiratory dynamics, evidenced by the decrease in VE, a main effect of condition on RR, and accumulation of blood CO_2_, demonstrating a relationship between changes in ventilatory patterns and changes in the metabolic profile caused by ARMs. In the present study, the magnitude of changes in the ventilatory response was not sufficient to significantly alter SaO_2_, and the variables relating to acid-base balance were the most strongly affected.

Studies that use large dead space volumes for RMT found changes in the ventilatory pattern and accumulation of blood CO_2_ [18]. These changes may be due to an increase in acidosis and alterations in the composition of the inspired air [18]. The dead volume of the ARM set (adapted mouthpiece and mask) used in the present study was approximately 350 ml, and a small dead space combined with the use of ARM valves may have altered the composition of the air in the mask. There were changes in the expired concentrations of O_2_ and CO_2_, and the CE-ARM condition presented a decrease in PETO_2_ and an increase in PETCO_2_. These results indicate that the air in the dead space had a higher concentration of CO_2_ and a lower concentration of O_2_, which may have increased blood CO_2_ concentration due to rebreathing of the expired air, as postulated previously [7,8].

The ARM may be used as a RMT device, this evidence was confirmed by changes in the ventilatory pattern (VE and RR) via a decrease in air volume, indicating the increased difficulty to perform the respiratory cycle and decreased RR, and demonstrating that time under tension of the respiratory muscles are increased. Additionally, ARM induced respiratory acidosis by increased CO_2_ rebreathing; a mechanism used in another RMT devices that use large dead space [18].

A relevant finding of this study was the increase in O_2_ consumption in the CE-ARM condition, characterized by high $\dot{V}O_{2}$ values. Given the lack of increase in electromyographic activity from SUM QF, we hypothesized that the higher consumption of O_2_ was due to the higher activity of respiratory muscles, which might decrease ventilatory efficiency because of the lower number of breaths. This phenomenon was been reported in previous studies that have used prolonged expiration as RMT [11].

### Limitations

The idea of the project was to evaluate the effect of wearing the mask on a population of physically active individuals. In this sense, adopting a mixed group did not seem to compromise the results. Other studies that aimed to evaluate the effects of using an implement or supplementation also adopted similar methodological approach [19]. A detailed overview of male and female analysis can be found in the supplementary material S1–S4 Tables.

Additionally, the present study has the following limitations. First, blood gas analysis indicated systemic alterations but not metabolic changes in the muscle, so a muscle specific analysis such as from muscle biopsies or even NIR-S could give us a better picture of muscle metabolism. Secondly, ventilatory muscles were not evaluated directly; therefore, the higher activation of these muscles is speculative. Finally, it is possible to speculated a potential additive ergogenic effect due any supplements taken by subjects, however as any supplementation was maintained during the entire study, any effect would occur equally in both conditions.

We recommend that future researchers investigate the relationship between the use of the mask and a heightened activation of the inspiratory musculature (through electromyography or inspiratory force assessments). Future studies should take into account the increased intensity of the exercise protocol, in order to exacerbate the physiological variations with the use of the mask. In conclusion, our results demonstrate that the use of ARMs did not cause hypoxia. Moreover, changes in VE affected metabolic parameters suggesting that the use of ARMs during exercise represents an additional challenge for the control of acid-base balance, therefore the use of ARM for RMT may be appropriate.

## Supporting information

S1 Table Separate man and woman characterization. (DOCX)

S2 Table Separate results of lactate for Man (M) and Woman (W). (DOCX)

S3 Table Mens gasometric values in CE and ARM. (DOCX)

S4 Table Women gasometric values in CE and ARM. (DOCX)

S1 Dataset Data used for statistical analysis. (XLSX)
