# Correction: The effect of an airflow restriction mask (ARM) on metabolic, ventilatory, and electromyographic responses to continuous cycling exercise

**DOI:** 10.1371/journal.pone.0248237

**Published:** 2021-03-02

**Authors:** The PLOS ONE Staff

## Notice of republication

An incorrect version of Supporting information file S1 Dataset was published in error. The publisher apologizes for this error. This article was republished on February 22, 2021 to correct for this error. Please download this article again to view the correct version.
