# Supplementary material for: The effect of an airflow restriction mask (ARM) on metabolic, ventilatory, and electromyographic responses to continuous cycling exercise
Source: PLoS One. 2020 Aug 11;15(8):e0237010. doi: 10.1371/journal.pone.0237010 (PMC7418989; doi:10.1371/journal.pone.0237010)
Supplement: S1 Table — (DOCX) [file pone.0237010.s001.docx]

| **S1 Table. Separate Man and Woman Characterization** | | | | |
| --- | --- | --- | --- | --- |
| **Incremental test** | **Man** | **Woman** | **Δ%** | **P-value** |
| Total Time (sec) | 1169.36 | 1041.83 | 10.9 | 0.01 |
| Work (w) | 277.27 | 220.83 | 20.3 | 0.001 |
| $\dot{\text{V}}$O_2_max (ml/kg/min) | 48.22 | 34.25 | 28.9 | 0.001 |
| $\dot{\text{V}}$O_2_max (ml/min) | 2462.91 | 1925.17 | 21.8 | 0.001 |
| $\dot{\text{V}}$CO_2_max (ml/min) | 3276.36 | 2655.67 | 18.9 | 0.01 |
| HR (bpm) | 184.2 | 185.17 | 0.5 | 0.88 |

$\dot{\text{V}}$O_2_max - Maximum oxygen intake; $\dot{\text{VC}}$O_2_max - Maximum carbon dioxyde output; HR – hart rate. The p value represent the T-test comparison between Man x Woman.

In the characterization variables, acquired in an incremental test, we can observe different between men and women, except for heart rate. This difference was expected, given the inherent differences in physical capacity between men and women.
