# Supplementary material for: The effect of an airflow restriction mask (ARM) on metabolic, ventilatory, and electromyographic responses to continuous cycling exercise
Source: PLoS One. 2020 Aug 11;15(8):e0237010. doi: 10.1371/journal.pone.0237010 (PMC7418989; doi:10.1371/journal.pone.0237010)
Supplement: S2 Table — (DOCX) [file pone.0237010.s002.docx]

| **S2 Table. Separate results of lactate for man (M) and woman (W)** | | | | | | | | |
| --- | --- | --- | --- | --- | --- | --- | --- | --- |
|  | **CE** | | | | **CE-ARM** | | | |
| **Time** | **M** | **W** | **Δ%** | **P-value** | **M** | **W** | **Δ%** | **P-value** |
| pre | 1.68 | 1.62 | 3.57 | 0.84 | 1.57 | 1.47 | 6.3 | 0.68 |
| post | 8.05 | 6.33 | 21.3 | 0.29 | 8.10 | 6.21 | 23.3 | 0.18 |
| 3 min | 7.38 | 5.90 | 20 | 0.41 | 7.32 | 6.22 | 15 | 0.45 |
| 5 min | 6.72 | 4.22 | 37 | 0.12 | 7.16 | 5.21 | 27.2 | 0.11 |
| 7 min | 5.94 | 4.69 | 21 | 0.24 | 7.07 | 5.06 | 28.4 | 0.17 |

M- Man; W- Woman; CE- Continuos Exercise; CE-ARM- Continuos Exercise with Mask. The p value represent the T-test comparison between M x W.

Although the variables analyzed in the incremental test show great difference between men and women, the lactate values in the pre, pos, three min, five min and seven min moments showed no differences, either in the condition with the mask (ARM) or in the condition without the mask (CE)
