# Supplementary material for: The effect of an airflow restriction mask (ARM) on metabolic, ventilatory, and electromyographic responses to continuous cycling exercise
Source: PLoS One. 2020 Aug 11;15(8):e0237010. doi: 10.1371/journal.pone.0237010 (PMC7418989; doi:10.1371/journal.pone.0237010)
Supplement: S3 Table — (DOCX) [file pone.0237010.s003.docx]

| **S3 Table. Mens gasometric values in CE and ARM** | | | | | | | | | |
| --- | --- | --- | --- | --- | --- | --- | --- | --- | --- |
|  | **CE** | | | **CE-ARM** | | | | **P-value** | |
|  | **pre** | **post** | **Δ%** | | **pre** | **post** | **Δ%** | **pre** | **post** |
| pH | 7.41 | 7.32 | 1.2 | | 7.42 | 7.28 | 1.8 | 0.79 | 0.01* |
| pCO_2_ [mmHg] | 36.8 | 35.4 | 3.8 | | 35.3 | 36.5 | -3.3 | 0.12 | 0.49 |
| pO_2_ [mmHg] | 84.7 | 80.2 | 5.3 | | 82.5 | 79.2 | 4 | 0.21 | 0.57 |
| Hct [%] | 44.8 | 47.6 | -6.2 | | 44.2 | 50.9 | -15.1 | 0.32 | 0.03* |
| SO_2_ [%] | 97.9 | 96.6 | 1.3 | | 98.1 | 96.1 | 2 | 0.65 | 0.18 |
| cHCO_3_ [mmol/l] | 23.1 | 18.0 | 22 | | 22.2 | 17.0 | 23.4 | 0.11 | 0.36 |
| BE [mmol/l] | -1.0 | -7.1 | 610 | | -1.7 | -8.8 | 417 | 0.23 | 0.12 |
|  |  |  |  |  | |  |  |  |  |

Means significant difference in T,test comparation, CE- continues Exercise; CE-ARM- continuous Exercise with Mask. The p value represent the comparison between pre x pre and post x post.

For the blood gas analysis variables in men, significant differences for the comparison between the ARM and CE conditions at the post moment was identified for the pH and gasometric variables, It was demonstrated that the ARM condition demanded more from the buffer system.
