# Supplementary material for: The effect of an airflow restriction mask (ARM) on metabolic, ventilatory, and electromyographic responses to continuous cycling exercise
Source: PLoS One. 2020 Aug 11;15(8):e0237010. doi: 10.1371/journal.pone.0237010 (PMC7418989; doi:10.1371/journal.pone.0237010)
Supplement: S4 Table — (DOCX) [file pone.0237010.s004.docx]

| **S4 Table. Women gasometric values in CE and ARM** | | | | | | | | | |
| --- | --- | --- | --- | --- | --- | --- | --- | --- | --- |
|  | **CE** | | | **CE-ARM** | | | **P-value** | | |
|  | **pre** | **post** | **Δ%** | **pre** | **post** | **Δ%** | **pre** | **post** |  |
| pH | 7.4 | 7.3 | 1.35 | 7.4 | 7.3 | 1.3 | 0.39 | 0.02* |  |
| pCO_2_ [mmHg] | 32.4 | 33.5 | -3.3 | 33.3 | 39.1 | -17.4 | 0.54 | 0.01* |  |
| pO_2_ [mmHg] | 86.0 | 81.5 | 5.2 | 82.3 | 81.6 | 8.5 | 0.43 | 0.99 |  |
| Hct [%] | 41.2 | 43.8 | 6.3 | 43.6 | 45.2 | 3.6 | 0.10 | 0.12 |  |
| SO_2_ [%] | 98.4 | 97.2 | 1.2 | 98.4 | 96.3 | 2.1 | 0.90 | 0.03* |  |
| cHCO_3_ [mmol/l] | 20.8 | 17.9 | 13.9 | 21.0 | 18.8 | 10.4 | 0.86 | 0.06 |  |
| BE [mmol/l] | -2.7 | -6.7 | 148 | -2.6 | -7.2 | 176 | 0.95 | 0.44 |  |

Means significant difference in T,test comparation, CE- continuous Exercise; CE-ARM- continuous Exercise with Mask. The p value represent the comparison between pre x pre and post x post.

The gasometric variables analysis in women reveals greater disturbances in the buffering system than in men, an indication that the stress on the system was greater for this group, The verifiable that present changes are in relation to the post moment, for the ARM x CE comparison, The use of the mask led to a greater drop in pH, as in men, increased CO_2_ pressure, a drop in O_2_ saturation was observed, and the protonated hemoglobin increased, Although women did not show higher lactate values, it seems that exercise was more intense for them, when the gasometric variables were analyzed.
